# Observations on Croup

**Published:** 1847-05

**Authors:** Alexander H. Stephens

**Affiliations:** President of the College


*Observations on Croup*—A paper read before the Fellows of the College of Physicians and Surgeons, by ALEXANDER H. STEPHENS, M.D., President of the College.

The frequent occurrence of croup, and its not unfrequent fatality in the northern and maritime regions, especially those of the United States, render important every addition to our knowledge of the nature and treatment of this formidable disease.

Up to the time of Dr. Bayley, of New York, no modern writer appears to have entertained correct pathological notions of this malady. It had previously been confounded with anginose affections of the fauces. It was, however, known to Hippocrates, who describes it in these remarkable words: "Ab angina homo suffocatur, oculi affecti sunt, ac velut strangulatis prominent; facies et fauces incenduntur, imo etiam collum intumescitur, vero nihil mali habere videtur."

We owe to the late Dr. Hosack of this city, the best description of the various stages of croup, and probably the best practical directions for the treatment of it. Yet, there are important points both of pathology and practice which he leaves wholly untouched, and others in which, if I am not mistaken, he is inaccurate.

It is usual among the medical men of this city, to speak of genuine croup, meaning that in which a membrane is formed in the trachea, and of spasmodic croup, many of them believing that inflammation either does not exist at all in the latter species, or that it is not the prior or primary morbid condition. These views I hold to be erroneous, and if carried out in practice, highly dangerous.

Professor Ware, of Boston, (the most recent writer on croup,) has recently presented another view on the subject, in a well reasoned paper, wherein he records numerous cases and dissections, showing how little that it is truly valuable to the American physicians in relation to croup, is to be found in European publications, more especially among the continental writers, or rather, how far they fall short in establishing those rules of practice by which alone, the American physician can successfully contend with the formidable malady. I am led to infer that it may present itself under different aspects in different regions. Be this as it may, the division of croup, proposed by Professor Ware, into four species, viz: catarrhal, membranous, inflammatory, and spasmodic, does not accord with my own experience, or with that of the most sagacious and experienced practitioners in this city, with whom I have conversed on the subject.

The forms under which croup has presented itself to my observation in this city, during a period of more than thirty years, are the following:

1. A child with coryza and occasional cough of the ordinary character, as in bronchitis, is playing about without sore throat, or redness of the fauces, or glandular swelling. He appears more than usually animated, his countenance, especially his eye, is unusually bright, and his mind exhilarated. His skin at this time is not heated during the day, but rather harsh to the feel and dryer than natural. To an acute observer with a nice ear, his voice will be a little sharper than usual, and if he cries for a time, the peculiar inspiration will excite alarm. On the second or third night the attack of croup commonly comes on after a few hours sleep, the symptoms being a ringing cough, hoarse inspiration, and great roughness of voice. If the patient dies, a membranous formation is found in the trachea, and more or less in the bronchial tubes. This is what all admit to be genuine inflammatory croup.

2. Without any noticeable illness whatever, a child suddenly wakes up in the night with spasmodic suffocating cough of the peculiar croupy sound, the same inspiration as in the former case, and the same hoarseness of voice. A drink of some kind is given; the next cough is less sonorous, but the croupy symptoms as before described remain. The case is usually relieved by an emetic and some stimulating application to the throat, both of which are kept for that purpose in almost every well regulated family in the city, where there are many children under eight years of age. If not so relieved, the patient may die within twenty-four hours or less, or after a lapse of two or three days, or even a week. Where the disease terminates quickly in death no well formed false membrane is seen, but only mucus in the trachea more or less thick, and redness about the glottis. This is the form to which the term spasmodic croup has been given. Spasm of what? Of the glottis undoubtedly, and from what cause? From the presence of vitiated secretions, and undigested decomposed food in the stomach, it is answered, and how does this act? By sympathy? Now, this cannot either be proved or even rendered probable. It is true, when the stomach empties itself by vomiting, the symptoms, for a time at least, and often permanently are relieved, but vomiting does more than unload the stomach. It relaxes the system, reduces the action of the heart, determines the fluids to the skin, which possesses so remarkable an antagonism to the mucous surfaces —above all it induces a copious secretion from the fauces, and thereby unloads the congested vessels of the glottis. It is admitted that an acid state of the stomach often causes irritation in the pharynx, which thence extends to the posterior part of the upper portion of the larynx. In adults this is beyond all doubt, and in children it is every way probable. Is the impression of these acrid matters, eructated, from the stomach or secreted in the pharynx, under particular circumstances, upon the larynx the cause of the sudden occurrence of croup? It would be difficult absolutely to disprove these propositions. In my mind they are not improbable, but on the other hand, admitting the connexion between disordered stomach and croup established as it is by the most extended observation, may it not be attributable in part, at least, to the fact that continued coldness of the surface is precisely the condition which fits the system, as well in childhood as in age, for the action of cold and moisture in producing inflammatory diseases?

But, setting aside these considerations, and under any view of the subject, what is the morbid condition of the glottis which gives rise to the croupy symptoms? If from cold it is inflammation, if from acrid secretions acting for more than a few minutes, it is and can be nothing else. There is, therefore, no spasmodic croup, if by spasm it is intended to exclude inflammation as a cause of that spasm.

But I am asked again how are the two kinds of croup above described to be explained pathologically. The answer to this query will appear in the classification of the forms of croup now proposed.

Under the term croup, properly so called, are included two affections, which may exist either separately or together.

1. The cynanche trachealis or trachitis, in which membranous exudation is more or less formed in the trachea before any affection of the larynx, and more especially of the glottis, takes place.

2. The cynanche laryngea or laryngitis or glottitis, in which the laryngeal or spasmodic symptoms occur first or exteriorly.

3. Between these two there are varieties of combination, and these constitute the great majority of the cases met with in actual practice. In the most pure case of the so called spasmodic croup, no practitioner can say beforehand that no fatal inflammation of the glottis will occur, or that no obstruction of the trachea by false membrane or solid mucus is to be apprehended.

Is the disease croup a specific disease? Is there any peculiarity in the inflammation which gives rise to that secretion in the trachea? Let us look to anatomy and physiology, and the observation of disease, and to dissections for answers to this question.

In the first place, between the most firm tubular form of false membrane, and inspissated mucus, and mucus of an ordinary consistence, we see in dissection of croup every grade and variety. If specific, its character should be more marked.

When a child attempts to swallow hot water, the membranous exudation is produced in the posterior fauces and upper part of the larynx. Here then is an ordinary cause of inflammation producing what some consider a peculiar and specific secretion.

This question has a bearing upon practice, because it is contended by some that the specific effect of mercury is the proper remedy for this specific secretion.

It remains for those who deny the specific character of the tracheal secretion to account for its existence there, rather than in the larynx and trachea. In the larynx it is more rarely met with, in the trachea it gradually becomes less tenacious, and more resembles ordinary inspissated mucus. May it not be merely inspissated mucus in all cases? mucus inspissated by rapid disiccation? If a portion of mucus is left in the trachea, the increased rapidity of respiration, and the narrow calibre of the tube, must necessarily remove its watery particles in a doubly augmented ratio; less so in the trachea, because the same volume of air in proportion to surface does not pass by, and the air also is more charged with the moisture in its previous passage through the trachea—less so in the larynx, because that tube is larger. Rarely is the membrane seen upon the glottis, because death arises from spasm ere it has time to form on that irritable part. Rarely in adults, because in them the trachea is double the size it is even in advanced childhood, and because they exert a stronger volition to detach by hawking the first tenacious mucus that is adherent to the trachea.

The surface of the trachea is very unirritable. Where foreign bodies enter by accident, as when a tube is forced into it by an artificial opening, no coughing is induced unless by its rising up the glottis is touched. A small foreign body has been known to remain for years, quietly lodged in one of the ventricles of the larynx. The trachea and the comparatively unirritable parts, are those in which inflammation may be going on for a considerable length of time, without exciting any very marked symptoms. This consitutes the true explanation of the two modes of invasion in croup.

Besides these three forms of idopathic, primary, or true croup, the laryngeal, the tracheal, and the mixed—there are forms of secondary croup, such as occur in measles, scarlet fever, and more especially in the malignant ulcerated sore throat, the diphtherite of Bretonneau. This last occasionally occurs sporadically with us, and is, I apprehend, very generally the disease which, under the term croup, carries off in quick succession two or more children in the same family. I have treated it successfully with calomel and opium, followed by wine whey, in conjunction with nitrate of silver to the throat—but my experience is too limited for me to assume to instruct others in regard to its nature and treatment. The French writers do not appear to discriminate between this affection and croup, as known here and in great Britain.*

Before speaking of the proper medical treatment, I will say a few words on a point of Hygiene.

1st. What is the best method of bringing up children, with a view to their exemption from this disease?

Two systems are adopted for this purpose—one is to allow free exposure and exercise in the open air, except in the very worst weather. The children being well guarded with warm clothing, are not suffered to cease their exercise until they re-enter the house. The second is to confine them within doors, during the whole of the winter, and the early part of the spring. My observation leads me to think that although the first plan, if it is followed with great care, is the best, yet the second is more easily pursued, and upon the whole is the safest.

2d. Under what circumstances should especial precautions be taken, with a view to ward off the attack?

A child between the ages of two and five years with catarrh and cough, however slight and unfrequent, is a fit subject for croup, and if that disease is prevailing at the time, an attack after any exposure to cold and moisture, or any excess in eating, is always probable. The child should be confined to the house and dieted.

The treatment of croup should be prompt and decided, for if left to itself, the disease would probably in general prove fatal. But although prompt and decided treatment is necessary, it does not follow that heroic treatment is always, or even generally required. But the existing symptoms must always be met by remedies adquate to subdue them. The great skill of an experienced practitioner is shown in determining what amount of active treatment is *essential* in any given case; how much is requisite to remove the threatening symptoms, and to induce a favorable change, and how soon he must recur to the more severe remedies, after the disease has been for a time meliorated.—*N. Y. Annalist.*—*Buffalo Medical Journal.*

